# Phyllodes Tumors of the Breast in 2026: A Contemporary Narrative Review of Diagnosis, Surgical Margins, Prognosis, and Emerging Therapies

**DOI:** 10.7759/cureus.118070

**Published:** 2026-10-17

**Authors:** George Mpourazanis, Dimitrios Alefragkis, Angeliki Siafaka, Iliana G Lialiou, Marios Theofanis Valakostas, Christos Poptsis, Pantelina-Danai Korkontzelou, Gabriel Dahan, Georgia Christodoulou, Rüdiger Schulz-Wendtland, Aikaterini Ntaflou, Panagiotis Tsirkas, Ioannis Korkontzelos, Ioannis Kosmas

**Affiliations:** 1 Department of Obstetrics and Gynecology, General Hospital of Ioannina G. Hatzikosta, Ioannina, GRC; 2 Second Department of Critical Care, Attikon University Hospital, National and Kapodistrian University of Athens, Athens, GRC; 3 Department of Nursing, School of Health Sciences, National and Kapodistrian University of Athens, Athens, GRC; 4 Department of Surgery, General Hospital of Ioannina G. Hatzikosta, Ioannina, GRC; 5 Department of Obstetrics and Gynecology, General University Hospital of Patras, Patras, GRC; 6 Department of Gynecology and Obstetrics, University Breast Center for Franconia Erlangen, University Hospital, Friedrich Alexander University of Erlangen-Nuremberg, Erlangen, DEU; 7 Department of Gynecological Radiology, Breast Center of Rottal-Inn Hospital, Eggenfelden, DEU; 8 Department of Anesthesiology, General Hospital of Ioannina G. Hatzikosta, Ioannina, GRC

**Keywords:** artificial intelligence and phyllodes tumors of the breast, core needle biopsy, management of phyllodes tumor, molecular pathology, phyllodes tumors of the breast, radiotherapy

## Abstract

Phyllodes tumors of the breast are rare fibroepithelial neoplasms characterized by significant heterogeneity regarding morphology, biological behavior, and clinical outcome. This review summarizes current developments in the diagnosis, histopathological and molecular classification, therapeutic management, and emerging technologies associated with phyllodes tumors of the breast. A literature search was conducted across PubMed/MEDLINE, Scopus, Embase, and Google Scholar, focusing on publications from the 2000-2026 period. The literature search covered publications from January 1, 2000, to September 30, 2026, and was last updated on September 30, 2026. Mammography, ultrasound, and magnetic resonance imaging contribute to preoperative assessment, although significant diagnostic overlap with fibroadenomas persists. Core needle biopsy is a key diagnostic tool, yet lesion heterogeneity can limit the accuracy of histological classification. Advances in molecular pathology have identified mutations and pathways associated with the progression and aggressiveness of phyllodes tumors, though these have not yet replaced morphological assessment. Surgical excision with negative margins remains the primary therapeutic approach, while the risk of local recurrence depends on histological grade, surgical margins, and other clinicopathological characteristics. Radiotherapy in phyllodes tumors of the breast appears to offer benefits primarily regarding local control in selected high-risk cases. Emerging technologies, including artificial intelligence, radiomics, and digital pathology, are proving to be promising tools for diagnosis, grading, and prognostic assessment. Overall, modern management of phyllodes tumors of the breast is shifting toward more personalized and multifactorial approaches.

## Introduction and background

According to the scientific literature, phyllodes tumors (PTs) of the breast are uncommon fibroepithelial neoplasms accounting for approximately 0.3% to 1% of all breast tumors and 2.5% of fibroepithelial lesions, with the remaining 97.5% represented by fibroadenomas [1]. These tumors are biphasic fibroepithelial neoplasms consisting of both neoplastic stromal components and a non-neoplastic epithelial component. The histological feature is an intracanalicular, leaf-like architecture, with stromal protrusions forming cleft-like spaces lined by bilayered epithelium composed of luminal and myoepithelial cells. The World Health Organization (WHO) divides such tumors into benign, borderline, and malignant categories according to an integrated evaluation of stromal cellularity, stromal atypia, mitotic activity, tumor borders, stromal overgrowth, and related morphological characteristics [2-4].

The incidence of PTs in the global population is statistically reported to primarily affect women aged 45 to 49 years, with an estimated occurrence rate of 2 to 2.1 cases per million women. There are several clinical series where these fibroepithelial neoplasms can impact adolescents. Michael Co et al. performed a 15-year multicenter clinical study involving 465 patients with PTs. In this study, the reported ages of the affected women ranged from 12 to 86 years. Another study reported 115 women affected by malignant PTs, with ages >70 years. These studies reveal that this kind of neoplasms can affect elder women. It is estimated that about 60% to 70% of all cases are categorized as benign. Borderline neoplasms occur at a rate of 15% to 26%, and malignant neoplasms account for 8% to 20% [4-7].

PTs in clinical practice typically present as a palpable, painless breast mass that can grow rapidly. A case series by Hassouna et al. involving 106 patients identified a breast lump and rapid growth as major symptoms. Another study with 57 patients noted palpable mass and breast pain, while rapid growth can lead to breast asymmetry, skin stretching, or in rare cases, skin breakdown, ulcers, or bloody nipple discharge. A retrospective analysis by Mitus et al. with 295 patients found that distant metastases were 75.7% in the lungs, 18.9% in bones, 10.4% in the brain, and 5.4% in the liver [8-10]. Despite significant advances in diagnosis and histopathological classification, PTs continue to pose a major clinical challenge due to their heterogeneous biological behavior and the difficulty in accurately distinguishing preoperatively between benign, borderline, and malignant lesions. The clinical course can vary significantly among histological subtypes, ranging from localized tumors with a favorable outcome to aggressive forms characterized by local recurrence or distant metastases [6-8]. Furthermore, morphological overlap with fibroadenomas and the need to evaluate a combination of histological features continue to complicate the diagnostic process [2]. The recent 6th edition of the WHO classification reflects ongoing efforts to refine histological classification and prognostic stratification [4]. At the same time, advances in molecular pathology and digital technologies are opening up new avenues for research into PTs, specifically regarding more accurate diagnosis, prognosis, and more personalized therapeutic approaches.

This narrative review aims to provide a revised overview of PTs of the breast, underscoring relevant aspects in modern clinical practice. It also analyzes advancements in molecular pathology and emerging therapies, focusing on established published literature and projecting developments up to 2026 to create a medically applicable framework for management and future research directions.

## Review

Methods

The narrative literature review was conducted aiming to identify peer-reviewed studies related to modern PT of breast screening, diagnosis, molecular classification, systemic treatment, and the role of artificial intelligence (AI) and to provide an updated overview of the current literature. A literature search was performed using Google Scholar, Scopus, Embase, and PubMed/MEDLINE, focusing on peer-reviewed English-language articles published. The literature search covered publications from January 1, 2000, through September 30, 2026, and was last updated on September 30, 2026. The search strategy utilized various combinations of key terms related to “phyllodes tumor,” “breast,” “fibroepithelial lesion,” “surgical margin,” “local recurrence,” “radiotherapy,” “MED12,” “TERT,” “molecular pathology,” and “malignant phyllodes tumor.” Studies considered for inclusion included cohort studies, randomized controlled trials, original research articles, systematic reviews, and relevant contemporary clinical guidelines. Reference lists of key articles and recent guidelines were also screened manually to identify additional relevant publications. This article was designed as a review rather than a systematic review; therefore, no formal meta-analysis or quantitative synthesis was performed. A PRISMA (Preferred Reporting Items for Systematic Reviews and Meta-Analyses) flow diagram and formal risk-of-bias assessment were not undertaken. The literature was organized according to major contemporary clinical aspects of phyllodes breast tumor diagnosis, including screening, prognosis, and current existing therapies.

Imaging and the preoperative diagnosis

Mammography, Ultrasound (U/S), and Magnetic Resonance Imaging (MRI)

Mammographic lesions in the literature of PTs have been described to be indefinite and can be simulating benign lesions such as cysts and fibroadenomas. The appearance and shape of such lesions are precise, lobulated, dense masses that can have a lucent halo. Generally, these tumors do not have a diagnostic trait that separates them from fibroadenomas or distinguishes between benign, borderline, and malignant subtypes. On mammography inspection, they appear to be dense, round, or oval, lobulated masses with partially indistinct or well-circumscribed margins [11,12]. In an ultrasonography, PTs appear as a solid, hypoechoic oval with moderately blurred or well-circumscribed margins and approximately 50% are lobulated, commonly with posterior acoustic enhancement. Doppler ultrasound usually reveals an increased vascularization. It is described that in a malignant subtype, this neoplasm may be accompanied by cystic areas on imaging examination [13]. In MRI, PTs are characterized as oval, round lobulated nodules with well-defined margins and low signal intensity on T1-weighted images. In hemorrhagic areas, the signal may appear hyperintense, while T2-weighted images show heterogeneous hyperintense signals with slit spaces [14].

A 23.7-year retrospective cohort study by Farshid et al. yielded 30 women diagnosed with PT. Among them, 22 women with PT were found by mammography, and 26.7% were diagnosed as interval neoplasms. In eight cases, malignant PTs were missed by routine screening. These findings reflect that mammography alone cannot detect or differentiate PTs, especially malignant subtypes, because these tumors have a rapid growth [15]. A retrospective study by Duman et al. included 72 patients diagnosed with fibroadenomas using mammography, U/S, and MRI, with 70 patients diagnosed with PTs, which were frequently larger than 3 cm. This study reported that these imaging methods possibly distinguish PTs from fibroadenomas and support clinical evaluation [16]. A retrospective study of 16 histopathologically confirmed PTs using both mammography and ultrasonography reported that both imaging methods can improve the radiologist's diagnosis of PTs and can also distinguish them from fibroadenomas, although there is still some diagnostic overlap [17]. A retrospective study by Ma et al. involving 182 patients analyzed benign, borderline, and malignant PT diagnoses. The study's nomograph, which integrated clinical evaluation and MRI features, demonstrated an AUC of 0.795 (95% CI: 0.639-0.835), suggesting its potential to differentiate between benign and non-benign PTs before surgery [18].

Core Needle Biopsy (CNB)

CNB has been widely utilized since the early 1990s as a minimally invasive technique for preoperative histopathological diagnosis of breast lesions [19]. This method is considered the gold standard for the preoperative diagnosis of these lesions and plays a major role in characterizing them, significantly influencing clinician decision-making for treatment plans for PTs. However, it has false-negative rates of 25% to 30% and an underestimation rate of 18.8% to 37.5% [20]. The diagnostic accuracy varies because these lesions are characterized as heterogeneous and share substantial histological overlap with fibroadenoma [21].

A retrospective analysis by Zhou et al. examined 128 patients with PTs of the breast, revealing a diagnostic accuracy of 13.3%. The sensitivity for benign and borderline tumors was low, while specificity was high across all tumor categories. The study concluded that CNB has limited diagnostic reliability for PTs, emphasizing the need for clinicians to integrate CNB findings with histological, clinical, and imaging data for improved diagnosis [22]. Tariq et al. studied 166 cases of fibroepithelial lesions who had preoperatively diagnosed with CNB, reporting a diagnostic accuracy of 90.4% for PTs of the breast. The study emphasizes the importance of integrating clinical and radiological findings for improved clinical outcomes [23]. Rakha et al. highlighted the value of evaluating stromal characteristics by pointing out challenges in identifying fibroepithelial lesions from CNB due to intralesional heterogeneity and the morphological parallels between cellular fibroadenoma and PT [24]. According to Soleimani et al., of 180 fibroepithelial lesion-not-further characterized (FEL-NFC) lesions diagnosed via CNB, 15% were found to be benign PTs and 7% benign, malignant after surgical excision. The study advocates for a selective excision strategy for indeterminate FELs, noting that larger tumor size, heterogeneous ultrasound features, and stromal overgrowth correlate with a higher risk of significant PTs [25].

In a recent study by Salisbury et al., the diagnostic performance of CNB was evaluated in 455 fibroepithelial lesions. The findings indicated a 19.7% upgrade rate to PTs, with 7% from fibroadenomas. Postoperative CNB effectively stratified the lesions, resulting in low local recurrence (LR) for fibroadenomas and high LR for PTs. Indeterminate lesions presented significant diagnostic challenges and often required excision. The study concluded that CNB is a valuable tool for managing fibroepithelial lesions when used alongside clinical and neoplasm characteristics [26]. The literature describes complications of this method, including localized pain, bruising, and hematoma formation. Rarely, more major complications like pseudoaneurysm and pneumothorax may occur. A 2026 study reaffirmed that while minor complications occur, serious issues necessitating hospitalization are uncommon. The strong safety profile of CNB supports its application in assessing fibroepithelial breast lesions [27].

Histopathological classification and molecular pathology of PT of the breast

Tumors are classified as benign, borderline, or malignant based on criteria such as stromal cellularity, atypia, mitotic activity, tumor borders, and stromal overgrowth. Histopathological evaluation remains essential for the diagnosis and grading of PT. The updated WHO 6th edition emphasizes the importance of these morphological parameters in breast pathology (Table 1) [4].

**Table 1:** Current classification of phyllodes tumors of the breast subtypes HPF: high power field Adapted from Quinn et al. [4] under the Creative Commons Attribution 4.0 International License (CC BY 4.0).

Histological Feature	Benign	Borderline	Malignant
Tumor border	Well-defined	Well-defined, may be focally permeative	Permeative
Stromal cellularity	Cellular, usually mild, may be non-uniform or diffuse	Cellular, usually moderate, may be non-uniform or diffuse	Cellular, usually marked and diffuse
Stromal atypia	Mid or none	Mild or moderate	Marked
Mitotic activity	Usually low: <2.5 mitoses/mm^2^ (<5 mitoses/10 HPF)	Usually frequent: 2.5 to <5 mitoses/mm^2^ (5–9 mitoses/10 HPF)	Usually abundant: ≥5 mitoses/mm^2^ (≥10 mitoses/10 HPF)
Stromal overgrowth	Absent	Absent (or very focal)	Often present
Malignant heterologous elements	Absent	Absent	May be present

Accurate classification of core biopsy specimens is challenging due to morphological overlap between fibroadenomas and PTs, along with intra-tumoral heterogeneity and observer variability. Molecular research, including findings by Cani et al., indicates that MED12 mutations are prevalent across histological grades, while TP53, RB1, and NF1 alterations, along with high-level amplifications of EGFR and IGF1R, are mainly linked to malignant tumors [28]. Kim et al. reported that 70.6% of PTs exhibited TERT-promoter mutations, while 64.7% had MED12 alterations. Their study associated changes in RARA, ZNF703, SETD2, BRCA2, TSC1, PTEN, and RB1 with disease recurrence or aggression [29]. These results were supported by Piscuoglio et al., who observed that mutations in TP53, RB1, SETD2, and EGFR were primarily found in borderline and malignant tumors, while TERT alterations progressively increased from benign to borderline and malignant PTs, being confined to the stromal component [30]. Genomic profiling of malignant PTs reveals increased complexity, including frequent alterations in TP53, TERT, NF1, MED12, CDKN2A/B, and activation of several pathways such as RTK/RAS/RAF and PI3K/AKT/mTOR, alongside sporadic actionable kinase fusions [31]. The research by Rosenberger et al. reveals that genomic profiling may play a significant role in advanced disease, noting TERT-promoter alterations in 69.7% of 135 malignant PTs, along with actionable mutations in PIK3CA, PTEN, NF1, EGFR, and BRAF [32]. Lee et al. propose a comprehensive model of molecular progression, indicating that early MED12 alterations precede additional driver abnormalities such as TERT, TP53, and EGFR, despite the limited clinical validation of molecular classification [33]. Pang et al. conclude that PTs exhibit greater genomic complexity correlated with grade, suggesting that molecular testing might be particularly beneficial in diagnostically challenging situations, rather than solely relying on morphology [34]. Valenza et al. found that fibroadenoma-like areas and MED12 mutations were not significantly associated with recurrence in malignant PTs, suggesting that their prognostic value is still unclear, supporting this cautious approach [35]. Zhou et al. found that in malignant PTs, a Ki-67 level of 30% or higher independently predicts lower overall survival, suggesting that proliferation markers are significant for risk stratification [36]. Current evidence supports a morphology-first, molecularly informed approach, emphasizing that genomic changes enhance prognostic and diagnostic assessment without replacing skilled histopathological evaluation.

Surgical management and local recurrence of PTs of the breast

Surgical treatment is the primary cornerstone for managing PTs, focusing on effective local control and complete tumor excision without unnecessary extent of surgery. Breast-conserving surgery (BCS) is now recognized as a suitable option for PTs when negative margins and acceptable cosmetic results are achievable, challenging the old preference for mastectomy based solely on tumor histology [37]. A recent multicenter retrospective study by Kang et al. involved 183 patients with borderline and malignant PTs, focusing on procedures like BCS and mastectomy. The study found that achieving a surgical margin of <1 mm is preferable to routine wide excision in breast cancer surgery, as it is a significant independent predictor of local recurrence (HR 2.07, 95% CI 1.03-4.08; p=0.043). While adjuvant radiotherapy indicated a trend towards improved local recurrence-free survival, it did not reach statistical significance (HR 0.25, 95% CI 0.05-1.19; p=0.081) [38]. Sars et al. conducted a long-term cohort study involving 191 patients with a mean age of 46 years who underwent BCS and mastectomy with varying margin statuses for benign, borderline, and malignant tumors. BCS was the first treatment for 93.2% of the patients, and a local recurrence rate of 10.5% was observed over a median follow-up of 10.1 years. The study revealed no significant correlation between final surgical margin status and local recurrence, with rates of 9.7% for negative margins and 7.4% for positive margins (p=0.70). These results advocate for the feasibility of BCS and challenge the necessity of wider surgical margins to prevent local recurrence [39].

In a study of 921 borderline and malignant tumors, Bartels et al. reported five-year LR rates of 8.7% for borderline tumors and 11.7% for malignant tumors. Although the margin widths (0-1 mm vs. >1 mm) did not show significant differences, positive margins were associated with a higher LR risk (HR 3.0, 95% CI 1.6-5.6). The findings suggest that margin positivity is more clinically relevant than the specific width of negative margins [40]. Del Calvo et al.'s study on 117 patients highlights that 64 had margins ≥10 mm, 32 had narrow margins (<10 mm), and 16 had positive margins. Positive (HR 10.57) and narrow margins (HR 5.66) are associated with a higher risk of local recurrence (LR) compared to wider margins. For borderline and malignant tumors, the 10-year LR rates were 93% with margins ≥10 mm and 57% with narrow margins. In benign tumors, a tumor-free margin on ink is considered sufficient for local control [41]. Ji et al.'s study of 238 patients emphasizes the importance of histological grade in surgical decision-making. It found that LR rates were significantly higher after limited excision compared to wide local excision or mastectomy for borderline and malignant tumors. However, no significant differences in LR were observed for benign PTs based on surgical method (P=0.397). Overall LR rates were 14.6% for benign, 34.2% for borderline, and 44.8% for malignant PTs, reflecting the aggressive biological behavior associated with higher histological grades [42]. Rodrigues et al. reported an overall LR rate of 8.7% in a cohort of 183 patients over a median follow-up of 65 months. The five-year LR rates were 6%, 9%, and 21% for benign, borderline, and malignant tumors, respectively. Margin status significantly influenced LR, with rates of 8% for negative margins, 6% for close margins, and a marked increase to 37% for positive margins (P<0.001) [43]. All relevant studies are mentioned in Table 2 [38-43].

**Table 2:** Εvidence at surgical management, margins, and local recurrence of phyllodes tumors LR: Local recurrence; BCS: Breast-conserving surgery; WLE: Wide local excision; LE: Local excision; N/A: Not available Table created by the authors based on data from references [38-43].

Study	Type of Study	Number of Patients	Mean Age (Years)	Type of Operation	Surgical Margins	Grade	LR	Follow-up (Median Months)
Kang et al. (2026) [38]	Multicenter retrospective study	183	N/A	BCS and mastectomy	<1 mm	Borderline and Malignant	LR: 0.25 with operation and radiotherapy	56
Sars et al. (2025) [39]	Multi-institution cohort study	191	46	BCS and mastectomy	Negative and positive	Benign, Borderline, and Malignant	Benign LR: 7.6%, Borderline LR: 17.5%, Malignant: 5.6%	121
Bartels et al. (2024) [40]	Original article	921	55	BCS and mastectomy	>1 mm positive margins	Borderline and Malignant	Borderline LR: 8.7% and Malignant LR: 11.7%	115.2
Calvo et al. (2024) [41]	Single-Institution retrospective cohort	117	44	BCS and mastectomy	>10 mm positive margins	Benign, Borderline, and Malignant	LR: 10.57 for all subtypes	85
Ji et al. (2022) [42]	Retrospective cohort study	238	N/A	WLE, LE, and mastectomy	WLE: >1 cm, LE: ≤1 cm	Benign, Borderline, and Malignant	N/A	50.2
Rodrigues et al. (2017) [43]	Retrospective population-based cohort	183	N/A	LE and mastectomy	Negative: 135, Positive: 20	Benign, Borderline, and Malignant	Benign LR: 6%, Borderline LR: 9%, Malignant LR: 21%	65

A recent systematic review and meta-analysis yielded by Harris et al. reviewed 13 studies with 1,658 patients having malignant or borderline PTs. It found that while mastectomy reduced local recurrence risk, BCS offered better overall survival without affecting disease-free survival. The authors concluded that BCS, when feasible, should remain a viable option, advocating for a tailored, multidisciplinary approach over routine mastectomy [44].

Radiotherapy in PTs of the breast

Adjuvant radiotherapy in PTs remains controversial and should be individualized according to histological subtype, tumor size, margin status, and the local recurrence risk. Boutrus et al. examined adjuvant radiotherapy in 108 patients with PTs. They found that, for those with borderline or malignant tumors, radiotherapy significantly improved local control, resulting in a 90% five-year local recurrence-free survival compared to 42% for surgery alone. This benefit was more significant in patients who had BCS with negative margins, while mastectomy showed no such improvement. However, radiotherapy did not enhance overall survival. The study suggests radiotherapy may reduce LR in specific cases of borderline and malignant PTs, particularly after BCS, rather than providing a survival advantage [45]. A retrospective study by Charoenyothakun et al. examined the impact of RT in 102 patients with borderline or malignant PTs. While there was no significant overall reduction in LR, malignant tumors indicated improved control recurrence was 12.2% in irradiated patients versus 36.4% without radiotherapy (P=0.081). A delay of over 12 weeks post-surgery for radiotherapy increased LR risk. These results imply potential benefits of adjuvant radiotherapy for high-risk malignant cases, though routine use cannot be advised due to the retrospective nature of the study and lack of statistically significant outcomes [46].

In a multicenter study of 480 patients with borderline and malignant PTs, postoperative radiotherapy (PORT) after R0 resection showed no significant improvement in five-year local recurrence-free survival (71.42% vs. 70.17%, P=0.85) or overall survival (82.41% vs. 80.02%, P=0.89) post-matching. No subgroup showed a benefit, suggesting that adjuvant radiation therapy should be tailored to individual clinicopathological risk factors rather than applied routinely after surgery, despite its potential for local control [47]. PORT significantly improved local recurrence-free survival in a retrospective study by Zheng et al. involving 108 patients, particularly benefiting intermediate and high-risk groups with larger tumors (>5 cm), positive lymph nodes, or undergoing BCS. However, radiotherapy did not enhance overall or distant metastasis-free survival, indicating it should be considered an individualized treatment option for patients at higher risk of local recurrence, rather than a standard treatment for all malignant PTs [48]. Present evidence suggests that radiotherapy should be used primarily as a selective local-control strategy for patients with malignant or high-risk borderline tumors, particularly when the risk of LR is high.

AI and radiomics in PTs of the breast

The use of AI is emerging as a promising supportive tool for healthcare professionals, particularly oncologists and surgeons, during the preoperative assessment of breast PTs. Specifically, ultrasound-based deep learning models have demonstrated the ability to distinguish PTs from fibroadenomas and to classify the tumors as benign, borderline malignant, or malignant [49,50]. More recently, the AI-FLEET study showed that multimodal approaches combining ultrasound imaging with clinical parameters yielded encouraging results in the classification of PTs [51], while radiomic analysis based on mammography and ultrasound may further enhance preoperative risk stratification [52]. AI-assisted analysis of whole-slide histopathology specimens has also been investigated as a potential aid for tumor staging [53]. Given the rarity of PTs, multi-center data sharing and the standardization of imaging and histopathological annotations will be crucial for improving the generalizability of future models [49,50]. Furthermore, explainable AI approaches may facilitate the clinical adoption of such tools by enabling radiologists and pathologists to better interpret model predictions [54]. However, current literature remains predominantly retrospective or limited to proof-of-concept studies; larger-scale studies including external validation of the validity and reliability of AI algorithms and models are required before their implementation in routine clinical practice becomes feasible. The current and emerging applications of AI in the diagnosis, grading, and clinical translation of PTs are summarized in Figure 1.

**Figure 1:**
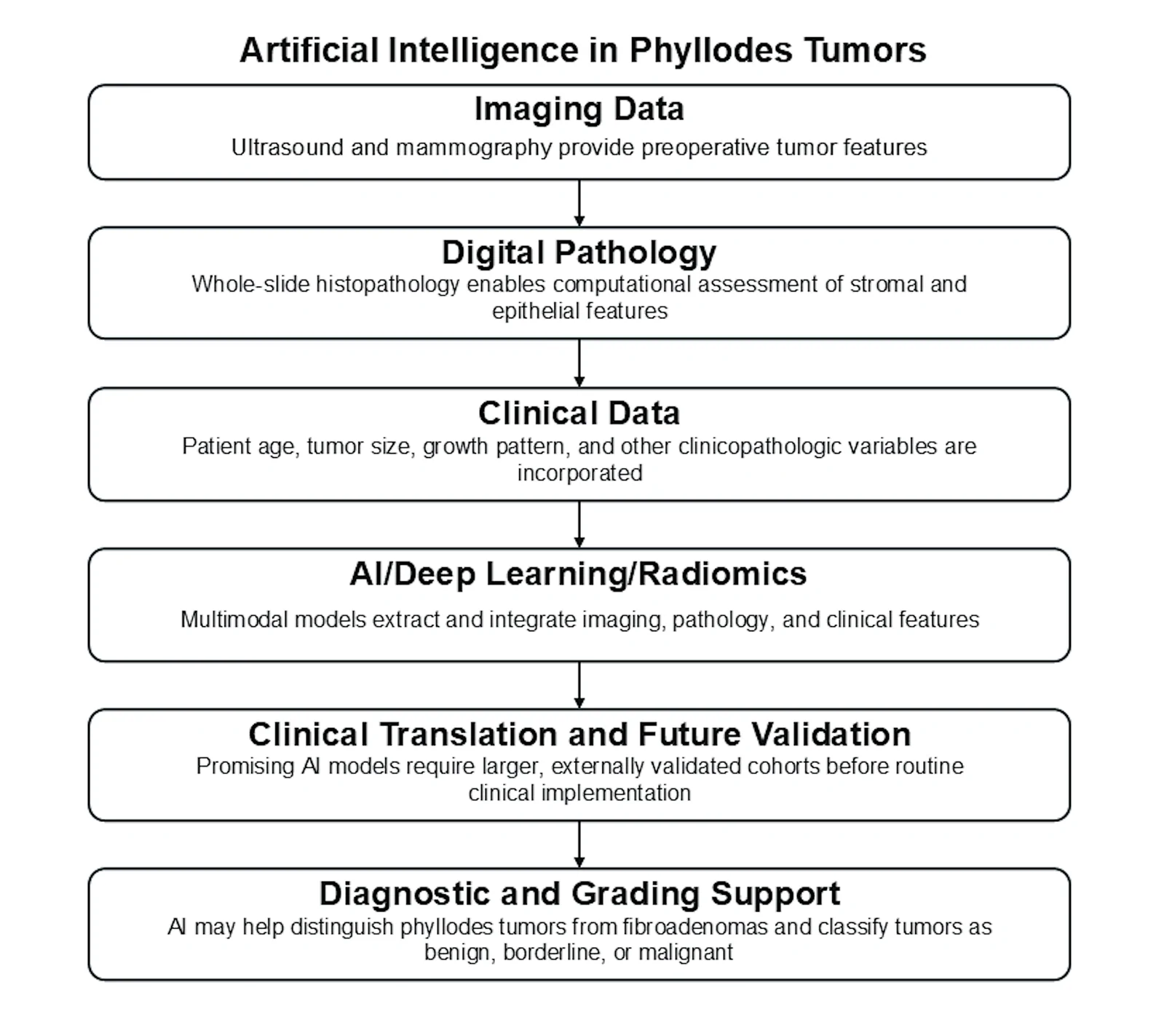
Emerging applications of artificial intelligence in phyllodes tumors The diagram summarizes the current and emerging applications of artificial intelligence in phyllodes tumors, including imaging-based analysis, digital pathology, integration of clinical variables, multimodal deep learning and radiomics, diagnostic and grading support, and the need for further external validation before routine clinical implementation.

Beyond ultrasound-based approaches, applications of AI and radiomics to PTs have expanded to include imaging modalities such as digital mammography, digital breast tomosynthesis (DBT), and MRI, as well as digital pathology and molecular data analysis. In a retrospective study of 192 patients, Zeng et al. developed radiomics models based on full-field digital mammography (FFDM) and DBT to differentiate PTs from fibroadenomas. The combined radiomics and radiological signature model achieved an AUC of 0.948 in the testing set and improved the diagnostic performance of radiologists [55]. In a more recent study, Su et al. evaluated mammography- and ultrasound-based radiomics in 362 patients with PTs, reporting a validation AUC of 0.791 for the nomogram model in distinguishing between benign and borderline or malignant tumors [52]. MRI has also been investigated for both diagnosis and pathological classification. Specifically, Liang et al. analyzed data from 77 patients using conventional radiomics, features of intra-tumoral sub-regions, and deep learning features derived from MRI, although data fusion models demonstrated high performance in the training cohort, AUC values ​​in the test set were 0.89 for differentiating PTs from fibroadenomas and 0.83 for pathological classification, thereby highlighting the need for larger external validation cohorts [56]. In a study by Ma et al., it was demonstrated that combined radiomics based on mammography and MRI could contribute to prognostic assessment, with a multimodal model achieving an AUC of 0.95 for recurrence-risk prediction, while the model also significantly stratified patients according to disease-free survival following surgery in 131 patients [57].

Digital pathology is another significant area of ​​research regarding PTs. Cheng et al. developed an AI model using whole-slide images from 187 fibroadenomas and 100 core biopsies of PTs, achieving an overall accuracy of 87.5% at the slide level in differentiating between the two entities [58]. More recently, Koong et al. investigated the histological classification of PTs using AI and whole-slide images; however, the study included only 15 cases and achieved a maximum accuracy of 67%, suggesting that it represents an early proof-of-concept rather than a clinically validated classification system [53]. At the molecular level, Gao et al. combined transcriptomic data with weighted gene co-expression network analysis (WGCNA), as well as Random Forest and LASSO regression methods, identifying CCL19 and CCR6 as potential biomarkers for distinguishing fibroadenomas from PTs, with internal ROC curve values ​​of 0.962 and 0.915, respectively [59]. Although recent large-scale genomic profiling studies have further characterized potential actionable molecular alterations in malignant PTs, the direct integration of genomic sequencing data into validated AI models remains limited [32,60]. In conclusion, these findings indicate that AI research on PTs is evolving from single-modality image analysis toward multimodal frameworks that integrate radiological, histopathological, clinical, and molecular information.

Future directions

The management of PTs is moving towards a more individualized approach, emphasizing the assessment of tumor biology and recurrence risk. While surgery remains the primary treatment, defining optimal surgical margins is critical, as current practices advocate for complete excision with negative margins but lack consensus on a fixed 1 mm margin. Future studies should explore how margin requirements vary based on factors such as histological grade and tumor size, while also weighing re-excision's risks against aesthetic and quality-of-life implications. Standardized pathological assessment is essential due to their heterogeneous nature and interobserver variability in grading. To improve consistency, criteria for evaluating stromal cellularity, atypia, mitotic activity, and margin status should be integrated. Molecular research highlights genomic complexities in malignant PTs, with alterations in pathways such as TERT, TP53, and PTEN. Future investigations should determine if these genetic changes can predict recurrence or metastasis risks, thereby refining treatment intensity for patients. AI and radiomics are emerging tools in clinical decision-making. Initial studies suggest their effectiveness in differentiating PTs from fibroadenomas and aiding histological grading, yet most models need validation across diverse settings. Ensuring AI systems integrate clinical and molecular data is key to enhancing their clinical utility and improving patient outcomes.

Due to the rarity of malignant PTs, international collaboration and multicenter registries are vital for advancing treatment in advanced disease, particularly for evaluating targeted therapies. The future of PT research should focus on personalized treatment strategies, leveraging histopathology, molecular data, imaging, and AI, to minimize unnecessary interventions and tailor therapy effectively.

## Conclusions

PTs of the breast remain a rare yet complex group of fibroepithelial neoplasms, characterized by significant heterogeneity regarding morphology, biological behavior, and clinical outcome. Accurate preoperative diagnosis continues to pose a major challenge, as imaging findings and core needle biopsy results may show considerable overlap with fibroadenomas and present limitations in reliably classifying histological grade. Surgical excision with negative margins remains the cornerstone of therapeutic management. However, current evidence supports a more personalized approach to the extent of resection, prioritizing factors such as surgical margin status, histological grade, and the risk of local recurrence rather than applying a uniform, predetermined margin for all patients. Concurrently, advances in molecular pathology and genomics are beginning to elucidate the biological basis of aggressive subtypes and highlight potential prognostic and therapeutic avenues. AI, radiomics, and digital pathology are emerging technological tools that may enhance the differential diagnosis, grading, and prognostic assessment of PTs. However, the majority of available models remain in the early stages of development and require external validation in larger, multicenter cohorts before implementation in routine clinical practice. Overall, the future management of PTs is expected to rely on a more comprehensive and personalized strategy incorporating a combination of histopathological, imaging, molecular, and clinical data. International collaboration, multicenter registries, and the standardization of pathological assessment can contribute significantly to improving diagnosis, recurrence-risk assessment, and therapeutic management, particularly for malignant forms of the disease.
